# Liver-specific deletion of RORα aggravates diet-induced nonalcoholic steatohepatitis by inducing mitochondrial dysfunction

**DOI:** 10.1038/s41598-017-16077-y

**Published:** 2017-11-22

**Authors:** Hyeon-Ji Kim, Yong-Hyun Han, Hyelin Na, Ju-Yeon Kim, Taewook Kim, Hye-Jin Kim, Chanseok Shin, Jung Weon Lee, Mi-Ock Lee

**Affiliations:** 1 College of Pharmacy, Seoul National University, Seoul, Korea; 2 Department of Agricultural Biotechnology, Seoul National University, Seoul, Republic of Korea; 3 Bio-MAX institute, Seoul National University, Seoul, Korea; 4 Research Institute of Pharmaceutical Sciences, Seoul, Republic of Korea

## Abstract

Mitochondrial dysfunction may play a key role in the progression of steatosis to nonalcoholic steatohepatitis (NASH); however, the molecular mechanism that controls the structure and function of mitochondria in NASH is not clearly understood. Here, we demonstrated that RORα is a regulator of expression of Bnip3 and PGC-1α, and thereby enhances mitochondrial quality. First, we observed that liver-specific RORα knockout mice (RORα-LKO) were more susceptible to high-fat diet-induced NASH compared with control, probably due to mitochondrial dysfunction. Concordantly, mitochondrial fission in response to nutrient stimuli was abolished with downregulation of Bnip3 and phospho-Drp1 in the hepatocytes of RORα-LKO. RORα enhanced oxygen consumption rate and expression of genes associated with mitochondrial quality control. Finally, we observed the positive correlation of the expression levels of Bnip3 and PGC-1α with those of RORα in patients with steatohepatitis. Together, we demonstrated that RORα mediates mitochondrial quality under nutrient-overloaded conditions and propose RORα as a potential therapeutic target in treatment of NASH.

## Introduction

Nonalcoholic fatty liver disease (NAFLD) ranges from simple steatosis to nonalcoholic steatohepatitis (NASH), which can eventually progress to irreversible cirrhosis and to hepatocarcinoma (HCC). About 10% to 20% of patients with hepatic steatosis develop NASH, a disease stage that is characterized by increased oxidative stress and lipotoxicity and leads to cellular injury and chronic inflammation^1–3^. However, the molecular mechanism underlying the development of NASH from steatosis remains unclear. The features that distinguish NASH from steatosis include defects in mitochondrial structure and function, as the mitochondria in the hepatocytes of patients with NASH are swollen and have an abnormal morphology with a loss of cristae, and the activities of mitochondrial respiratory complexes are impaired in these patients^4,5^. Thus, mitochondrial dysfunction is considered as a central cause of the development of steatosis to NASH, and the identification of the molecular mechanisms that control the structure and function of mitochondria will contribute to the understanding of the progression of NAFLD.

Mitochondria are the main organelles that are required for energy-yielding metabolism via the oxidative phosphorylation (OXPHOS) of glucose and lipids. Maintaining mitochondrial quality is critical for cellular survival, because mitochondria are frequently vulnerable to reactive oxygen species (ROS), a side product of electron transfer. Multiple quality control mechanisms contribute to the maintenance of mitochondrial activity and function, including biogenesis, fission, fusion, and mitochondria-selective autophagy (mitophagy)^6^. New mitochondria are generated by mitochondrial fission resulting from a fragmentation of the mitochondrial network, especially to reduce oxidative stress in nutrient-overloaded conditions. In contrast, mitochondrial fusion is stimulated by energy demand, to increase metabolic efficiency^7^. The core machineries of mitochondrial dynamics include the dynamin-1-like protein (Drp1), fission 1 homolog (Fis1), mitochondrial fission factor, BCL2/adenovirus E1B 19 kDa interacting protein 3 (Bnip3), mitofusin 1, 2 (Mfn1, 2), and optic atrophy 1 (Opa1)^8^. Mitochondrial biogenesis is regulated by a nuclear–mitochondrial network^9^. In this network, the peroxisome proliferator-activated receptor γ coactivator (PGC-1α) acts as a master regulator that modulates the activity and expression of nuclear respiratory factors, followed by the induction of the transcription factor A, mitochondrial (TFAM), which stimulates mitochondrial gene expression^10^. Nuclear receptors, such as the estrogen-related receptor (ERR) and peroxisome proliferator-activated receptor (PPAR), are also reported to play a role in connecting the nutrient influx status to mitochondrial gene expression^11^.

The retinoic acid receptor-related orphan receptor α (RORα) is an orphan nuclear receptor that is associated with the regulation of various genes in hepatic lipid metabolism and inflammation^12^. RORα binds to a specific DNA sequence, called ROR response element (RORE), consisting of the monomeric RGGTCA motif or Rev-DR2 sites of direct repeats^13,14^. Natural and synthetic ligands, such as cholesterol sulfates, SR1078, and JC1-40, reversibly bind to RORα and increase the transcriptional activity of target genes via the induction of the DNA binding of RORα and recruitment of coactivators such as p300 and PGC-1α^15–18^. In patients with NAFLD and animal NASH models, the hepatic expression levels of RORα are significantly decreased, suggesting that RORα may be associated with pathogenesis in NASH^19,20^. Recently, we demonstrated that RORα has an inhibitory effect on lipid accumulation and oxidative stress, thereby attenuating hepatic steatosis and NASH^15,19^. The protective function of RORα against NASH strongly suggests that RORα plays roles in mitochondrial quality control under the pathological condition of oversupply of nutrients.

The physiological functions of RORα have been studied mainly using the RORα-deficient staggerer mice (RORα^sg/sg^), which carry a C-terminal deleted form of RORα^21,22^. RORα^sg/sg^ mice fed a high-fat diet (HFD) are resistant to the development of hepatic steatosis and exhibit decreased fasting blood glucose levels and increased insulin sensitivity^23–25^. However, this animal model has limitations regarding liver studies, because the systemic expression of the staggerer gene leads to the development of pathological phenotypes, such as immunodeficiencies, osteoporosis, cerebellar degeneration, atherosclerosis, and muscular atrophy^21^. Moreover, homozygous RORα^−/−^ mice exhibit tremor and abnormal body balance and die between 24 and 28 days of life^26^. Thus, we generated mice with a liver-specific deletion of the RORα gene using albumin-*cre*, to study the hepatic roles of RORα in the progression of NASH. Here, we demonstrated that RORα is a potent regulator of mitochondrial quality control in the liver in response to metabolic input. In particular, Bnip3 and PGC-1α were the transcriptional targets of RORα in the regulation of mitochondrial fission and biogenesis.

## Results

### Liver-specific KO of the RORα gene enhances susceptibility to HFD-induced steatohepatitis

**Figure 1:**
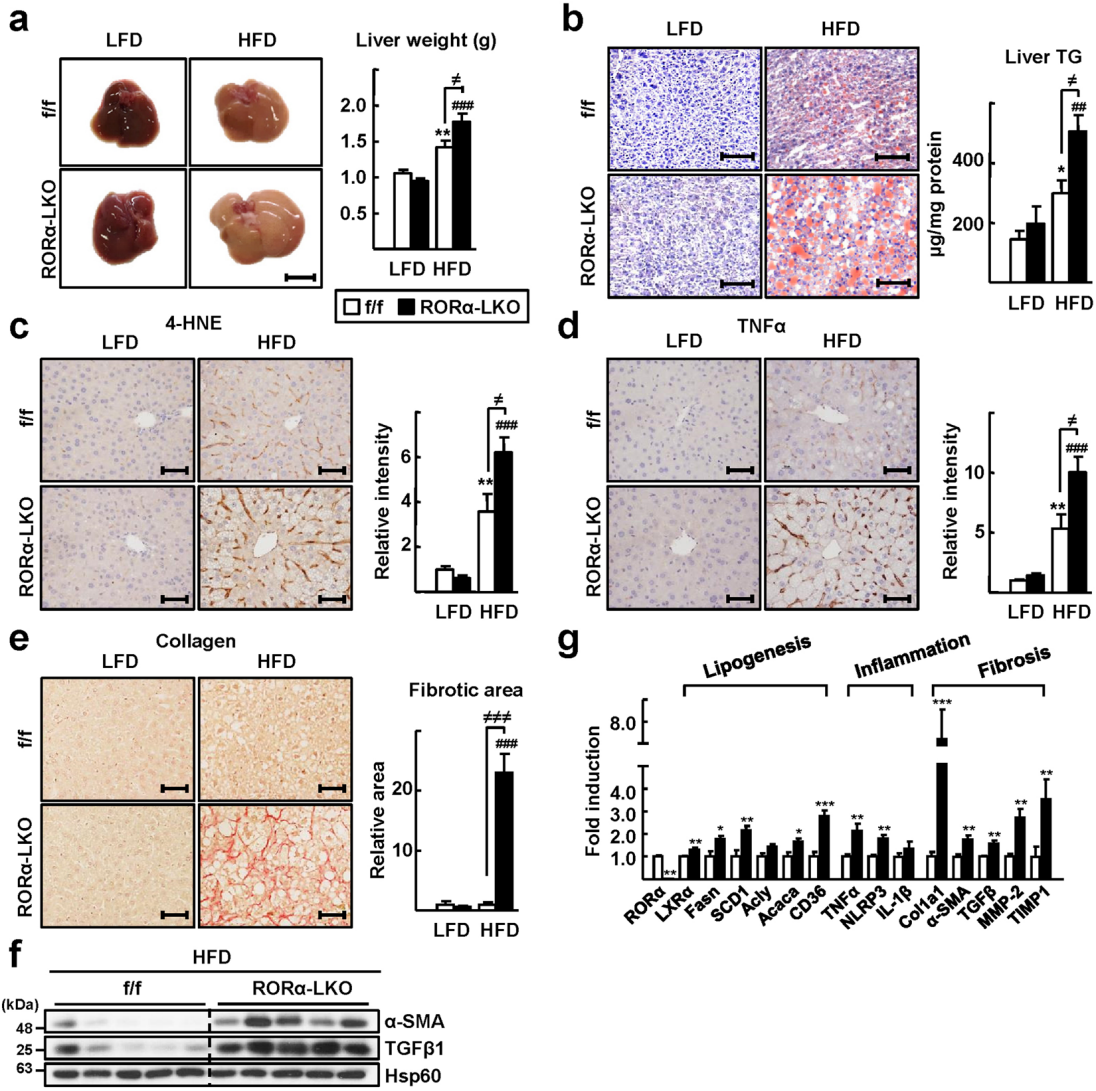
Liver-specific KO of RORα enhances susceptibility to HFD-induced steatohepatitis. (**a**) Six week-old RORα-LKO and RORα^f/f^ mice were fed with either LFD or HFD for 12 weeks. Representative images of livers and the liver weights of experimental mice at the end of experiments. Scale bar: 1 cm. Values represent mean ± SEM (n = 7–9). ^**^ *P* < 0.01 *vs* LFD-fed RORα^f/f^, ^###^ *P* < 0.001 *vs* LFD-fed RORα-LKO, ^≠^ *P* < 0.05 *vs* HFD-fed RORα^f/f^. (**b**) Oil red O staining of liver sections and hepatic TG levels. Scale bar: 100 μm. Representative images are shown. Values represent mean ± SEM (n = 7–9). ^*^ *P* < 0.05 *vs* LFD-fed RORα^f/f^, ^##^ *P* < 0.01 *vs* LFD-fed RORα-LKO, ^≠^ *P* < 0.05 *vs* HFD-fed RORα^f/f^. (**c**,**d**) Histological staining of 4-HNE (brown) and TNFα (brown). Scale bar: 50 μm. Representative Images of liver sections from the RORα^f/f^ and RORα-LKO mice were presented. Relative intensities were quantified using ImageJ. Values represent mean ± SEM (n = 6–8). ^**^ *P* < 0.01 *vs* LFD-fed RORα^f/f^, ^###^ *P* < 0.001 *vs* LFD-fed RORα-LKO, ^≠^ *P* < 0.05 *vs* HFD-fed RORα^f/f^. (**e**) Sirius red staining in the liver sections for detection of collagen deposition (red, left). Scale bar: 50 μm. Fibrotic area in the liver sections was analyzed using ImageJ (right). Values represent mean ± SEM (n = 6–8). ^###^ *P* < 0.001 *vs* LFD-fed RORα-LKO, ^≠≠≠^ *P* < 0.001 *vs* HFD-fed RORα^f/f^. (**f**) The protein levels of α-SMA and TGFβ1 were analyzed by western blotting in the liver tissues (n = 5). The original blots are shown in Supplementary Fig. S7. (**g**) The mRNA levels of factors related to lipogenesis, inflammation, and fibrosis were measured by qRT-PCR in the liver tissues. LXRα, Liver X receptor alpha; Fasn, Fatty acid synthase; SCD1, Stearoyl-CoA desaturase 1; Acly, ATP citrate lyase; Acaca, Acetyl-CoA carboxylase; TNFα, tumor necrosis factor alpha; NLRP3, NACHT, LRR and PYD domains-containing protein 3; IL-1β, Interleukin 1 beta; Col1a1, collagen Type I; α-SMA, alpha-smooth muscle actin; TGFβ, transforming growth factor β; MMP-2, matrix metalloproteinase-2, and TIMP1, TIMP metallopeptidase inhibitor 1. Values represent mean ± SEM (n = 7–9). ^*^ *P* < 0.05, ^**^ *P* < 0.01, and ^***^ *P* < 0.001 *vs* HFD-fed RORα^f/f^.

To study the hepatic role of RORα during the progression of NAFLD, we generated a liver-specific RORα-null mouse, RORα-LKO (Supplementary Fig. S1a,c). The absence of RORα gene expression was demonstrated in the liver of RORα-LKO mice, whereas the hepatic expression of RORβ or RORγ remained at control levels (Supplementary Fig. S1b). We fed an HFD to RORα-LKO mice for 12 weeks, to monitor the effects of deletion of the RORα gene on symptoms of NASH. Hepatic steatosis was severe in RORα-LKO mice compared with flox/flox (f/f) mice. The livers of RORα-LKO mice weighed more than did those of f/f mice, and the accumulation of lipid droplets was evident in the hepatocytes of the former (Fig. 1a,b). The indicators of liver injury, i.e., serum alanine aminotransferase (ALT), aspartate aminotransferase (AST), and the marker of lipid peroxidation 4-hydroxynonenal (4-HNE) were increased largely in the livers of RORα-LKO mice (Supplementary Fig. S2c, Fig. 1c). Consistently, hepatic expression of a proinflammatory cytokine, tumor necrosis factor alpha (TNFα), were increased in RORα-LKO mice (Fig. 1d). Also, the expression level of F4/80, a marker of infiltrated macrophages, was increased in the livers of RORα-LKO mice (Supplementary Fig. S2d). In addition, collagen deposition was increased in the liver tissues of RORα-LKO mice and the expression of alpha-smooth muscle actin (α-SMA) and transforming growth factor β1 (TGFβ1), pro-fibrotic factors, were significantly increased in the livers of RORα-LKO mice (Fig. 1e,f). The mRNA levels of genes involved in lipogenesis such as liver X receptor alpha and fatty acid synthase, inflammation such as TNFα and NACHT, LRR and PYD domains-containing protein 3 (NLRP3), and fibrosis such as collagen Type I (Col1a1) and α-SMA were significantly increased in the livers of RORα-LKO mice (Fig. 1g). Together, these data showed that the hepatic expression of RORα is closely associated with the development of NASH.

### Mitochondrial function is a target of hepatic RORα

Next, we carried out a transcriptomics study to identify the target genes of RORα associated with the development of NASH. Results from an RNA-seq analysis combined with the data from a public ChIP-seq analysis (GSE59486) revealed that a total of 2,639 genes were specifically altered by RORα (Supplementary Fig. S3a). A gene ontology (GO) analysis of the altered genes revealed that “oxidation-reduction” was the top-ranked GO biological process that was targeted by RORα. Interestingly, the GO term “electron transport chain” was the top GO biological process among the 134 genes in the cluster of oxidation-reduction (Supplementary Fig. S3b). Moreover, we found that the GO cellular component included mitochondrial components with statistical significance (Supplementary Fig. S3c). Surprisingly, the relative expression of most of the genes in the GO terms of “electron transport chain”, “carboxylic catabolic process”, and “ATP biosynthetic process” were significantly decreased in the livers of HFD-fed RORα-LKO mice, suggesting that mitochondrial function may be a target of RORα-mediated transcriptional regulation (Supplementary Fig. S3d).

**Figure 2:**
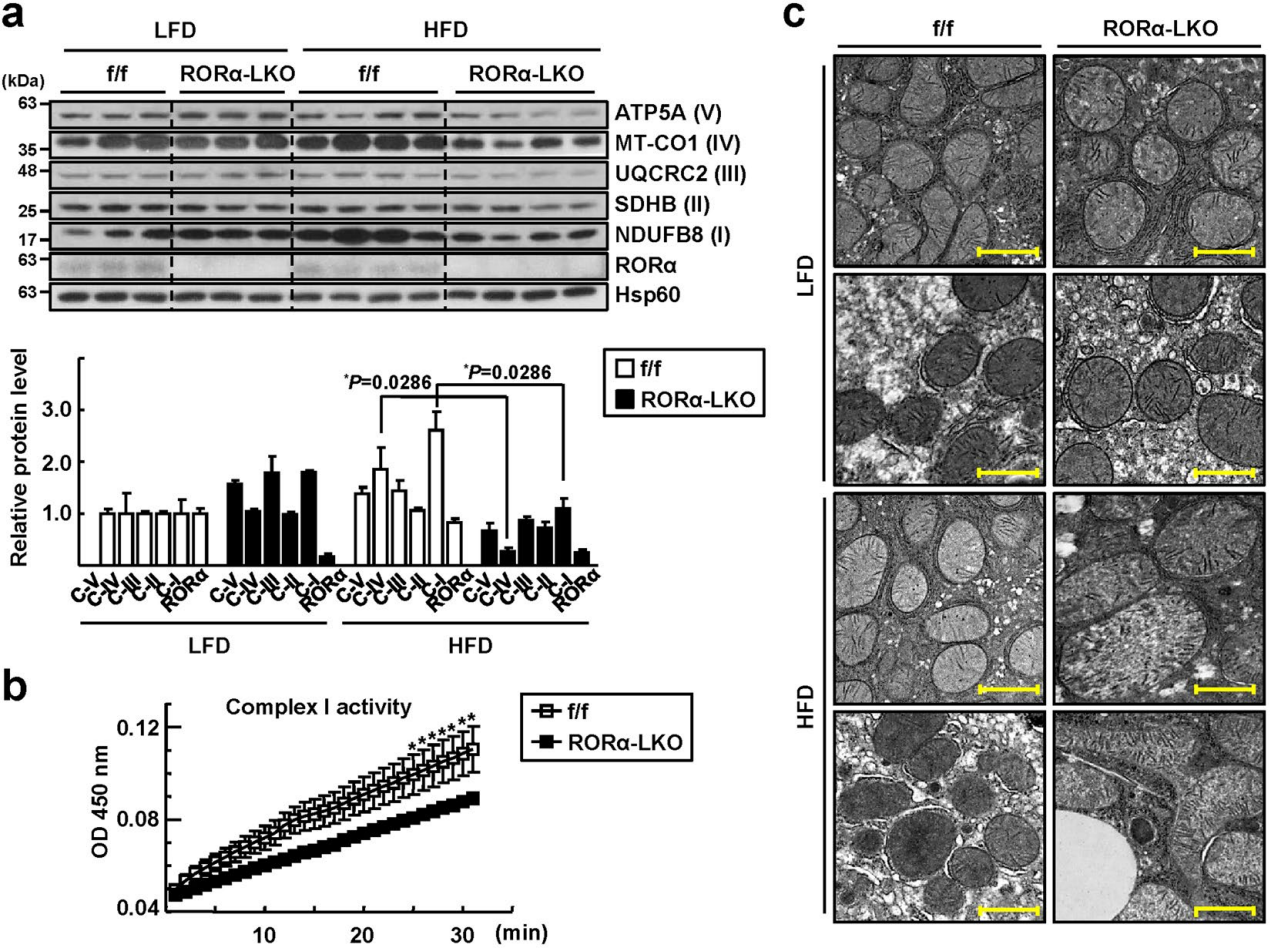
Mitochondrial defects in the liver tissues of RORα-LKO mice. (**a**) Hepatic levels of OXPHOS proteins in ETC complexes were analyzed by western blotting using a commercially available anti-total OXPHOS primary antibody cocktail. NDUFB8, NADH dehydrogenase [ubiquinone] 1 beta subcomplex subunit 8; SDHB, Succinate dehydrogenase [ubiquinone] iron-sulfur subunit; UQCRC2, Cytochrome b-c1 complex subunit 2; MTCO1, mitochondrially encoded cytochrome c oxidase I; and ATP5A, ATP synthase, H + transporting, mitochondrial F1 complex, alpha 1. Roman numbers represent the corresponding ETC complex. Band intensities of each protein were quantified using ImageJ and normalized to that of Hsp60 band. Data presented as mean ± SEM. ^*^ *P* < 0.05 *vs* HFD-fed RORα^f/f^ (n = 4). The original blots are shown in Supplementary Fig. S7. (**b**) Activities of complex I in the liver tissues from HFD-fed mice were measured by spectrophotometry based on the rates of NADH oxidation. Data presented as mean ± SEM. ^*^ *P* < 0.05 *vs* HFD-fed RORα^f/f^ (n = 3–4). (**c**) Representative EM images of the liver sections from RORα^f/f^ and RORα-LKO mice. Scale bar: 1 μm.

Therefore, we examined whether mitochondrial function was defective in the livers of RORα-LKO mice. First, we found that the expression of mitochondrial OXPHOS proteins, such as NADH dehydrogenase [ubiquinone] 1 beta subcomplex subunit 8 (NDUFB8), succinate dehydrogenase [ubiquinone] iron-sulfur subunit (SDHB), cytochrome b-c1 complex subunit 2 (UQCRC2), mitochondrially encoded cytochrome c oxidase I (MTCO1), and ATP synthase, H + transporting, mitochondrial F1 complex, alpha 1 (ATP5A), was decreased in the livers of HFD-fed RORα-LKO mice (Fig. 2a). Similarly, the activity of the mitochondrial complex I was decreased in the liver tissues of RORα-LKO mice (Fig. 2b). Moreover, we observed that mitochondria in the hepatocytes of RORα-LKO animals were deformed and showed an enlarged or swollen phenotype after HFD feeding (Fig. 2c). Together, these data showed that mitochondrial function may be a target of hepatic RORα.

### RORα is a novel modulator of mitochondrial fission in response to nutrient status

**Figure 3:**
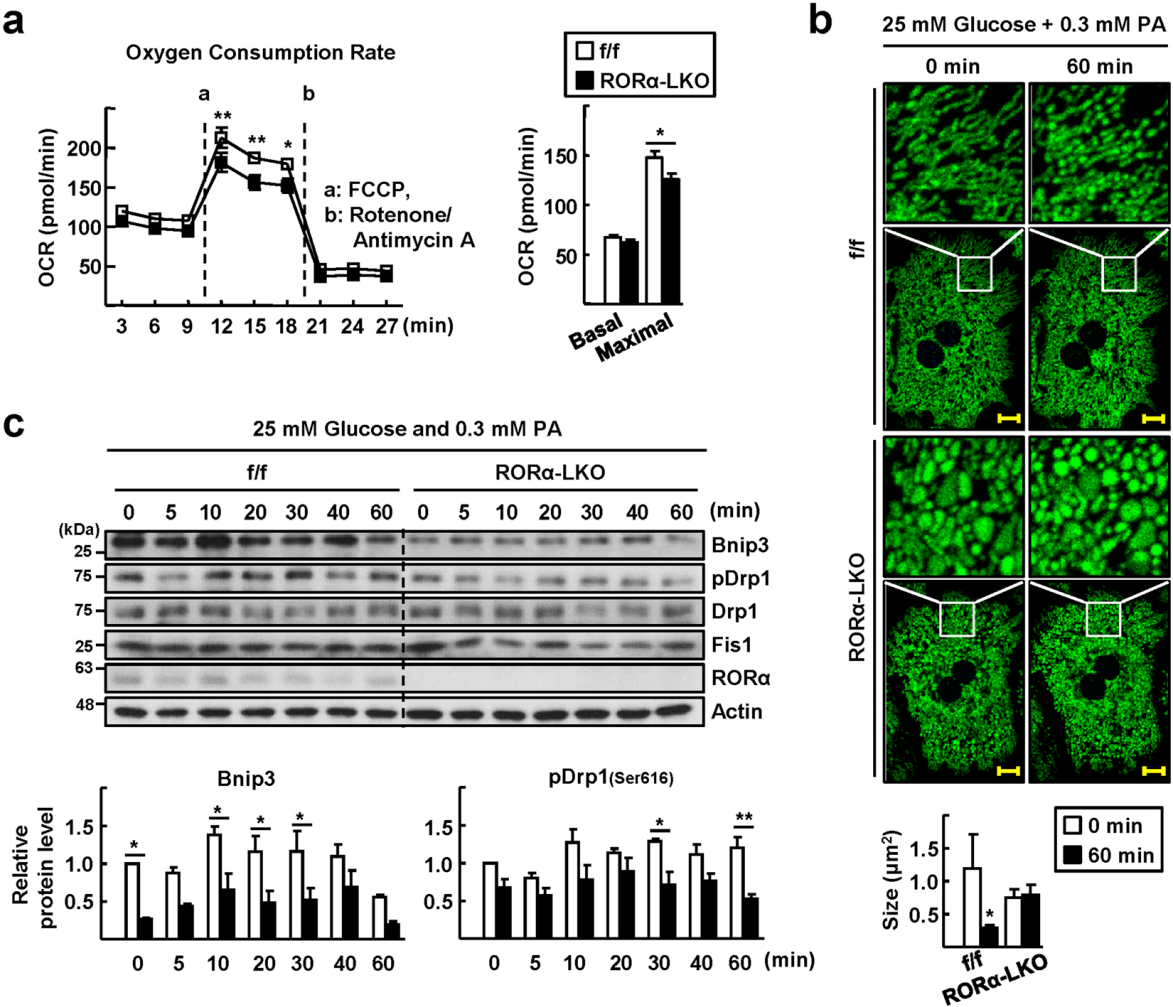
Mitochondrial fission is impaired in the hepatocytes of RORα-LKO mice. (**a**) The basal OCR and maximal respiration of control and RORα-LKO primary hepatocytes. a, b refer to the time course of adding FCCP, a inducer of maximal respiration, and antimycin A/rotenone, respectively (left). Data presented as mean ± SEM (n = 3). ^*^ *P* < 0.05 and ^**^ *P* < 0 0.01 *vs* RORα-LKO. The basal OCR, and maximal respiration were calculated based on data in left panel (right). Data presented as mean ± SEM. ^*^ *P* < 0.05 *vs* RORα-LKO. (**b**) Hepatocytes were infused by ad-COX8a-GFP to tag mitochondria for visualization. Hepatocytes were cultured in low nutrient condition (5.5 mM glucose) for 2 h, and then exchanged to the media containing 25 mM glucose and 0.3 mM palmitic acid. Photos were taken by real-time confocal microscopy. Representative time-lapse images of the mitochondrial morphology are shown (upper). Scale bar: 10 μm. The average mitochondrial size was quantified using ImageJ (lower). Data presented as mean ± SEM. ^*^ *P* < 0.05 *vs* 0 min (RORα^f/f^). (**c**) Cell lysates were obtained at the indicated time after media change and the levels of proteins associated with mitochondria dynamics were analyzed by western blotting (upper). Band intensities of Bnip3 and pDrp1 were quantified using ImageJ and normalized to that of actin (lower). Data presented as mean ± SEM. ^*^ *P* < 0.05 and ^**^ *P* < 0.01 *vs* RORα-LKO (n = 3). The original blots are shown in Supplementary Fig. S7.

Based on the observation of enlarged mitochondria in the livers of HFD-fed RORα-LKO mice, we hypothesized that the deletion of RORα causes defects in the process of mitochondrial fission, especially under conditions of nutrient overload. First, we measured the oxygen consumption rate (OCR) in hepatocytes isolated from RORα-LKO mice. Although the basal OCR was not much different between RORα-LKO and f/f control mice, the maximal OCR was significantly lower in RORα-LKO hepatocytes (Fig. 3a). To investigate mitochondrial dynamics further, we employed ad-COX8a-GFP to label mitochondria. The culture of primary hepatocytes from control mice in medium with low glucose (5.5 mM), which is a condition of energy demand, led to a hyperfused and elongated mitochondrial network. After the culture was challenged by high glucose (25 mM) with palmitic acid (300 μM), mitochondria were rapidly fragmented (Fig. 3b). However, the morphology and dynamics of mitochondria in RORα-LKO hepatocytes were largely different from those of control animals, in that mitochondria were swollen and remained not much different after the nutrient challenge (Fig. 3b). Along the time lapse after the high-nutrient challenge, the protein levels of Bnip3 and phospho-Drp1 (pDrp1), which are fission proteins, were decreased in RORα-LKO hepatocytes, whereas those of Fis1 was not (Fig. 3c). The decreases in Bnip3 and Drp1 levels were also observed in the liver tissues of HFD-fed RORα-LKO mice (Supplementary Fig. S4). Together, these data showed that mitochondrial fission in response to nutrient stimuli was abolished in RORα-LKO hepatocytes and suggest that the downregulation of Bnip3 and phospho-Drp1 might cause defects in mitochondrial quality control in RORα-LKO hepatocytes.

### Overexpression of RORα enhances mitochondrial dynamics via the induction of Bnip3 and pDrp1

**Figure 4:**
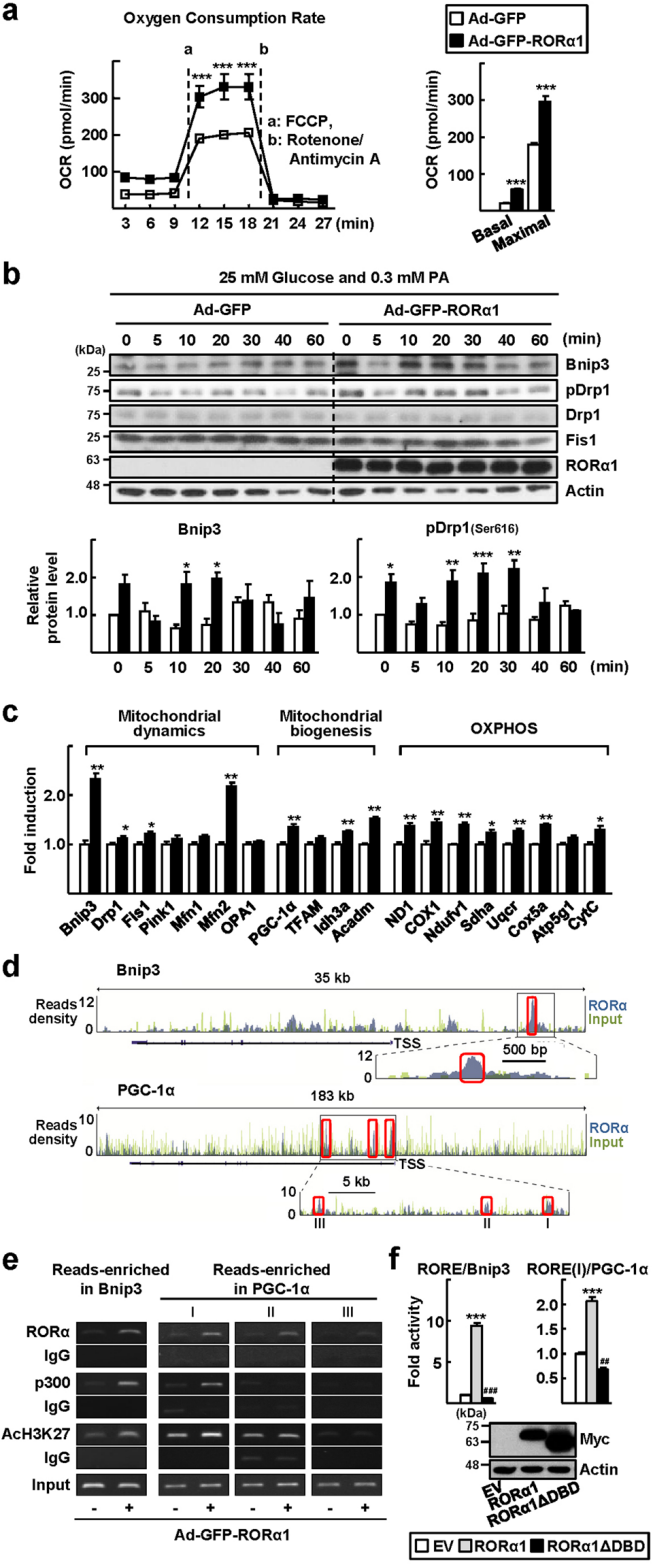
Overexpression of RORα enhances mitochondrial dynamic response. (**a**) The basal OCR and maximal respiration of primary hepatocytes infused by either Ad-GFP or Ad-GFP-RORα1. a, b refer to the time course of adding FCCP, and antimycin A/rotenone, respectively (left). Data presented as mean ± SEM. ^***^ *P* < 0.001 *vs* Ad-GFP infused hepatocytes (n = 3). The basal OCR, and maximal respiration (right). Data presented as mean ± SEM. ^***^ *P* < 0.0001 *vs* Ad-GFP infused hepatocytes. (**b**) Cell lysates were obtained at the indicated time after media change and the level of proteins associated with mitochondria dynamics were analyzed by western blotting (left). Band intensities of Bnip3 and pDrp1 were quantified using ImageJ and normalized to that of actin (right). Data presented as mean ± SEM. ^*^ *P* < 0.05, ^**^ *P* < 0.01, and ^***^ *P* < 0 0.001 *vs* Ad-GFP infused hepatocytes (n = 3). The original blots are shown in Supplementary Fig. S7. (**c**) After mouse primary hepatocytes were infected by either Ad-GFP or Ad-GFP-RORα1 for 18 h, the mRNA levels of factors related to mitochondrial dynamics, mitochondrial biogenesis, and OXPHOS were measured by qRT-PCR. The values represented as mean ± SEM. ^*^ *P* < 0.05 and ^**^ *P* < 0.01 *vs* Ad-GFP infused hepatocytes (n = 6). (**d**) The ChIP-seq reads of RORα and liver input control in the genome loci of Bnip3 and PGC-1α are shown in ChIP-seq tracks. Boxes indicate the regions that RORα signals are significantly enriched (Bnip3, chr7 146111103–146111273; PGC-1α I, chr5 51943784–51943954; PGC-1α II, chr5 51937131–51937301; PGC-1α III, chr5 51919125–51919295). A line below the ChIP-seq track represents transcript of the gene. TSS, transcription start site. (**e**) Primary hepatocytes were infused with either Ad-GFP or Ad-GFP-RORα1 for 18 h. DNA fragments were immunoprecipitated with the anti-RORα, anti-p300, or anti-histone antibodies and then amplified by PCR with specific primers. (**f**) Chang liver cells were transfected with the RORE/Bnip3-Luc or RORE(I)/PGC-1α-Luc with empty vector or the expression vector encoding Myc-RORα1 or Myc-RORα1 ΔDBD for 24 h (upper). The protein expression of Myc-RORα1 and Myc-RORα1 ΔDBD is shown (lower). The values represented as mean ± SEM. ^##^ *P* < 0.01 and ^***, ###^ *P* < 0 0.001 *vs* empty vector transfected cells (n = 3). The original blots are shown in Supplementary Fig. S7.

Finally, we confirmed the function of RORα in mitochondria quality control via the transient overexpression of RORα. Viral infusion of Ad-GFP-RORα1 into primary hepatocytes dramatically enhanced OCR at both the basal and maximal levels (Fig. 4a). The expression levels of Bnip3 and pDrp1 after nutrient overload were significantly higher in RORα1-overexpressing hepatocytes (Fig. 4b). Consistently, the mRNA expression of Bnip3, but not that of the PTEN-induced putative kinase 1, Mfn1, and Opa1, increased in the presence of overexpression of RORα1, indicating that Bnip3 is a potential target of RORα. In addition, the overexpression of RORα1 resulted in increases in the mRNA expression of genes associated with mitochondrial biogenesis, including PGC-1α, and oxidative phosphorylation (Fig. 4c). Indeed, data from the ChIP-seq analysis showed that RORα-binding signals were present on the regulatory regions of Bnip3 and PGC-1α (Fig. 4d). Additional ChIP assays confirmed that RORα bound to the reads-enriched regions in the Bnip3 and PGC-1α genes. Signals of transcription activation, such as the recruitment of p300 and the acetylation of H3K27, were clearly observed in the presence of RORα in some of these regions (Fig. 4e). Analysis of the reporter genes encoding the putative ROREs present in the regulatory regions of Bnip3 and PGC-1α showed that RORα induced transcriptional activities of the RORE/Bnip3-Luc and the RORE(I)/PGC-1α-Luc by 9-fold and 2-fold, respectively. However, the RORα1 ΔDBD, which lacked DNA binding domain, did not induce the reporters (Fig. 4f). These results indicate direct regulation of Bnip3 and PGC-1α by RORα.

### Expression levels of hepatic Bnip3 and PGC-1α were low and correlated positively with those of RORα in patients with steatohepatitis

**Figure 5:**
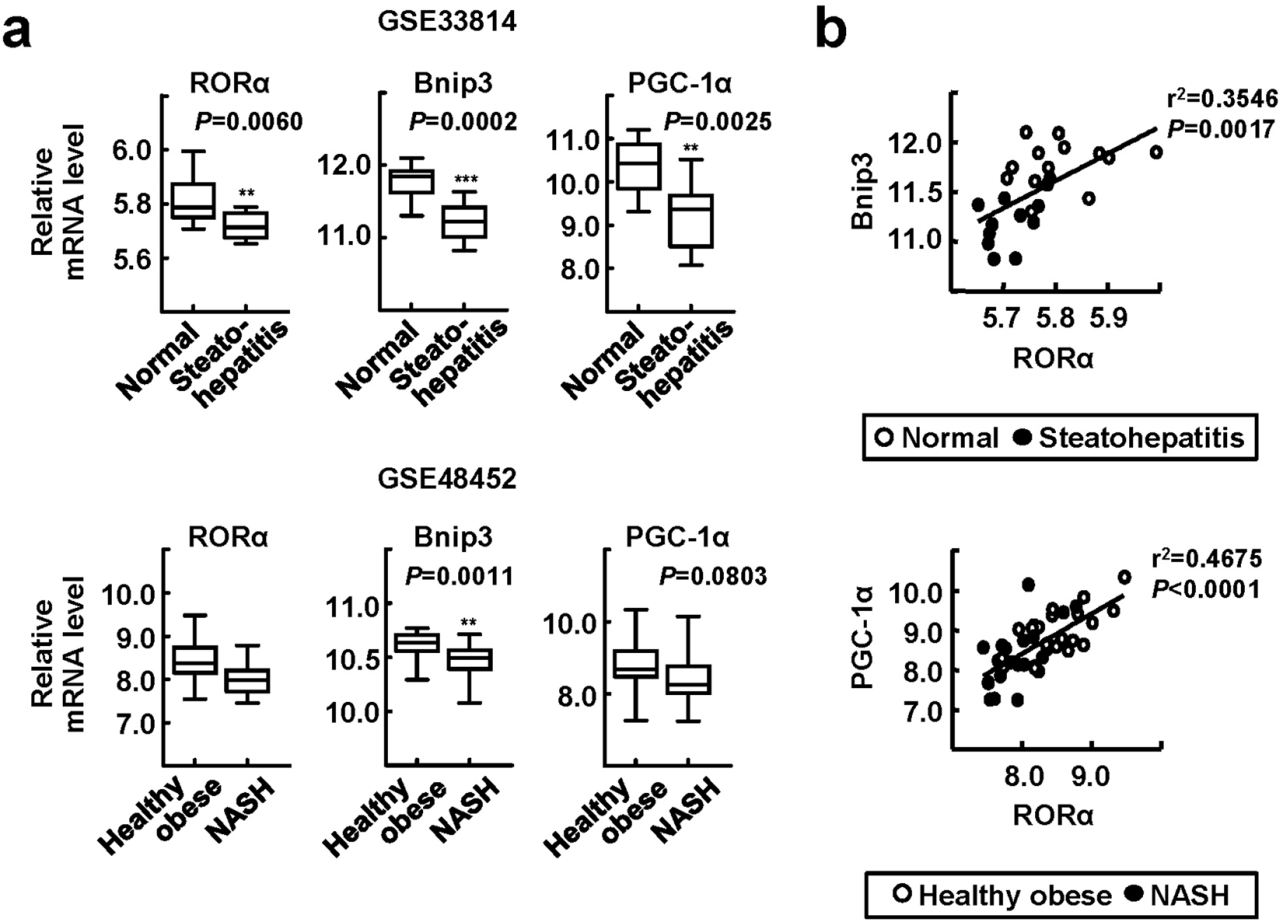
Expression levels of RORα, Bnip3, and PGC-1α in the livers of patients with steatohepatitis. (**a**,**b**) Database-based gene expression analysis was conducted using public datasets obtained from GEO site at the NCBI gene expression (http://www.ncbi.nlm.nih.gov/geo/). The data processed as median-normalized signal intensity value. Significances were analyzed by Mann-whitney U test and the positive correlation coefficient (r^2^) was calculated by Pearson correlation test. ^**^ *P* < 0.01, and ^***^ *P* < 0.001 vs normal or healthy obese (n = 13 (normal) and n = 12 (steatohepatitis) for GSE33814; n = 27 (healthy obese) and n = 18 (NASH) for GSE48452).

To assess the clinical relevance of our findings, we analyzed the expression levels of RORα, Bnip3, and PGC-1α in the livers of patients with steatohepatitis, including NASH, using publicly available databases (GSE33814 and GSE48452)^27,28^. As shown in previous reports, the expression level of RORα was diminished in patients with steatohepatitis^19,20^. Interestingly, the levels of Bnip3 and PGC-1α were also decreased in the livers of patients with steatohepatitis compared with those of healthy obese patients (Fig. 5a). The observation that the expression levels of Bnip3 and PGC-1α correlated positively with those of RORα suggests a potential application of our findings to diagnostic and therapeutic interventions for NASH (Fig. 5b).

## Discussion

Here, we report that the loss of the function of hepatic RORα resulted in the development of severe NASH in mice, which supports a protective role of RORα against the progression of NAFLD. The function of RORα in hepatic lipid metabolism has been controversial for some decades. RORα^sg/sg^ mice fed an HFD are resistant to diet-induced hepatic steatosis and insulin resistance^23,25^. In contrast, we found that RORα activates AMPK, while it represses the transcription function of LXRα, thereby protecting from hepatic lipid accumulation^15^. Moreover, RORα upregulated antioxidative and anti-inflammatory genes, which ameliorated the symptoms of NASH in the methionine and choline deficient diet mouse model^19^. Although the reasons for this discrepancy are not fully understood, the secondary effects generated by the sg/sg phenotype may contribute, at least in part, to this discordance in hepatic lipid metabolism. For example, the secretion of hormones that are important for energy balance, such as leptin, norepinephrine, adrenocorticotropic hormone, and corticosterone, was abnormal in RORα^sg/sg^ mice^25,29,30^. These mice were lean despite their hyperphagia, probably because of an enhancement of the energy metabolism in brown adipose tissue^29^. This imbalanced whole-body metabolism may cover or surpass the primary phenotype of RORα depletion in the liver; thus, the liver-specific RORα mice may provide a better animal model for studying the hepatic function of RORα. Recently, two research groups reported a protective role of RORα against hepatic steatosis by employing the liver-specific depletion of RORα or RORα/γ. They showed that loss of negative regulation of the peroxisome proliferators-activated receptor-γ or overactivation of the sterol regulatory element-binding proteins exacerbated diet-induced hepatic steatosis in these animals^31,32^. Here, we revealed a novel role of RORα in mitochondrial quality control that inhibits further progression of hepatic steatosis to NASH.

We found that Bnip3 is one of the major downstream effectors of RORα-controlled mitochondrial quality maintenance (Fig. 4c,d). Bnip3 is a well-known mitochondrial fission factor that promotes the translocation of Drp1 to mitochondria and simultaneously decreases Opa1-induced mitochondrial fusion^33^. Consistently, the depletion of Bnip3 in mice resulted in the loss of mitochondrial membrane potential and structural integrity, probably because of impairment of mitochondrial fission and subsequent mitophagy^34^. Interestingly, the loss of Bnip3 led to typical features of steatohepatitis, such as reduced AMPK activity and fatty acid β-oxidation and increased lipid synthesis, which were observed in the livers of RORα-LKO mice^34^. In human patients with NASH, the hepatic levels of Bnip3 were decreased compared with those of healthy obese individuals and correlated positively with the expression levels of RORα (Fig. 5a,b). Together, these observations strongly support our proposed mechanism of Bnip3-mediated RORα action. Bnip3 is also involved in the mitophagic process by interacting with the microtubule-associated protein 1 light chain 3 (LC3)^35^. Mitophagy is a cellular process triggered by severe mitochondrial defects that eventually promotes the survival of cells under stress conditions^6^. It was reported that the mitophagic flux is impaired in the livers of both NAFLD patients and murine models of NAFLD, which could be caused by an elevation of endoplasmic reticulum (ER) stress^36^. The results of our RNA-seq and ChIP-seq analyses showed that the ER ranked at the top of the GO cellular components for RORα-regulated genes (Supplementary Fig. S3c). In addition, RORα induced the mRNA levels of Mfn2, which is enriched at the ER–mitochondria interface and promotes intercommunication (Fig. 4c)^37^. These observations suggest that RORα mediates the mitochondrial quality control, which is linked to the regulation of ER maintenance.

The observation that RORα increased the expression of markers for mitochondrial biogenesis, such as PGC-1α, Idh3a, and Acadm, suggests that RORα may enhance mitochondrial biogenesis (Fig. 4c). However, total mitochondrial number was not affected by knock-down of RORα (Supplementary Fig. S5). One of the possible causes of this discrepancy may be that quantity of mitochondria is regulated by the balance of two opposing processes, *i.e*., mitochondrial biogenesis and elimination of mitochondria. Thus, no effect on the mitochondrial number by RORα could be due to the RORα-induced biogenesis and simultaneous elimination of mitochondria by RORα-induced Bnip3, one of the key factors involved in the mitophagic process^35^.

As mitochondrial dysfunction is associated with various human diseases, including Alzheimer’s disease, multiple sclerosis (MS), and retinal diseases, the roles of RORα in the pathophysiology of these diseases should be addressed. Acquaah-Mensah *et al*. (2015) reported that RORα expression was abnormal in the hippocampus of patients with Alzheimer’s disease, and that RORα was highly connected in a network of differentially expressed genes, which included genes involved in mitochondrial dynamics such as Fis1 and Opa1^38^. The pathobiology of MS, which is an autoimmune disorder of the central nervous system, is accompanied by mitochondrial dysfunction; the expression of nuclear-encoded mitochondrial genes and the activities of mitochondrial complexes were decreased in the MS motor cortex in patients with this disease^39^. Interestingly, polymorphism of intronic variations in the RORα gene was associated with susceptibility to MS^40^. Similarly, a significant link between single-nucleotide polymorphisms present in intron 1 of the RORα gene and a mitochondrial dysfunction-related disease, aged-related macular degeneration, was demonstrated using a systems biology-based approach^41^. Although there is no clear evidence currently, we suspect that RORα-mediated mitochondrial function might be associated with the development of these diseases. In addition, we found a potential that JC1-40, an agonistic ligand of RORα, improved mitochondrial function (Supplementary Fig. S6). Further studies of pharmacological interventions targeting RORα-mediated mitochondria quality control may provide therapeutic strategies against NASH as well as other diseases associated with defects in mitochondrial function.

## Methods

### Animal studies

The RORα^f/f^ mutant embryo, which has loxP sites flanking exon 4 of the RORα gene, was obtained from the Institut Clinique de la Souris (Illkirch, France) and the mutant mouse was generated by *in vitro* fertilization (Korea Research Institute of Bioscience and Biotechnology). To produce the liver-specific RORα KO line (Alb^Cre^-RORα^f/f^), RORα^f/f^ animals were crossbred with Alb^Cre^ animals, which express Cre recombinase in hepatocytes under the control of the albumin promoter (Jackson Laboratories). Several backcrosses of the two mouse lines produced the liver-specific RORα KO mice on the C57BL/6 background. Offspring were genotyped to confirm the inclusion of loxP sites within RORα alleles and the presence of Cre recombinase via PCR using specific primers (Supplementary Fig. S1, Supplementary Table). All animals were maintained with 12 h light (7 am) and dark (7 pm) cycles.

Six-week-old male RORα^f/f^ or RORα-LKO mice were fed an HFD (D12492) or low-fat diet (D12450J) (Research Diets, New Brunswick, NJ) for 12 weeks. HFD-fed RORα-LKO mice gained more weight compared with control mice (Supplementary information, Figure S2A). After feeding, liver tissues were excised and cross sections of the left lobe of the liver were analyzed for protein and mRNA, or fixed in 10% neutral buffered formalin (Sigma-Aldrich) for immunohistochemistry. The activities of ALT and AST in the serum were measured using a Fuji DRI-CHEM 3500 s serum biochemistry analyzer (Fujifilm, Japan), and the amount of hepatic TG was measured using an EnzyChrom^TM^ Triglyceride Assay Kit (BioAssay Systems). For histological examinations, 3 μm sections of paraffin-embedded tissue were stained with hematoxylin and eosin (H&E). Frozen liver tissue sections were stained with Oil red O staining. All experiments were performed in a blinded and randomized fashion. The experimental protocols were approved by the Seoul National University Institutional Animal Care and Use Committee (permission number SNU-140424-2-5) and all experiments were conducted according to the committee’s guidelines.

### Determination of OXPHOS protein expression and complex I activity

OXPHOS proteins in ETC complexes were analyzed by western blotting using a commercially available anti-total OXPHOS primary antibody cocktail (458099, Invitrogen, 1:20000). Proteins were extracted from liver tissues and quantified by bicinchoninic acid assay (Pierce). After quantification, we added sample buffer and let the samples stand for 30 min at 37 °C, for the detection of mitochondrially encoded cytochrome c oxidase I (MTCO1) proteins. An anti-Hsp60 antibody (ab45134, Abcam, 1:10000) was used as a control and the intensity of western blots was quantified by ImageJ. Complex I activity was measured using the Complex I Activity Assay Kit (AAMT001-1KIT, Novagen). Proteins were extracted from liver tissues by adding detergent, and each diluted sample (100 μg protein/200 μl) was added into a well coated with a monoclonal antibody against the NADH dehydrogenase complex. Complex I activity was determined based on the rate of NADH oxidation, which is linked to the reduction of a dye, leading to increased absorbance at 450 nm (according to the manufacturer’s protocol).

### Electron microscopy

Liver tissues were excised and small blocks from the left lobe were fixed in 2.5% glutaraldehyde in 0.1 M phosphate buffer (pH 7.0). The blocks were postfixed with osmium tetroxide, followed by En bloc staining with 0.5% uranyl acetate. After samples were dehydrated with 30%, 50%, 70%, 80%, 90%, and 100% ethanol, they were embedded in Spurr’s resin and polymerized. Ultrathin sections were cut using an EM UC7 ultramicrotome (Leica, Germany), and examined by JEM1010 transmission electron microscope (JEOL, Japan).

### Assessment of oxygen consumption rate

Primary hepatocytes were isolated from 8–10-week-old male C57BL/6 mice via the perfusion of livers with collagenase type IV (Sigma-Aldrich), as described previously^19^. After perfusion, cells were suspended in Dulbecco’s modified Eagle’s medium (DMEM) (Hyclone) containing 10% fetal bovine serum (FBS). The oxygen consumption rate (OCR) was analyzed using the Seahorse XFp Extracellular Flux Analyzer. Mouse primary hepatocytes isolated from control and RORα-LKO mice were plated in assay plates at 10^4^ cells/well with minimal DMEM (XF base medium) supplemented with 10 mM pyruvate, as described previously. In the case of assessment of the OCR of primary hepatocytes infected with either Ad-GFP or Ad-GFP-RORα, hepatocytes were plated in DMEM with 10% FBS 4 h prior to transduction. After 18 h, media were exchanged to unbuffered minimal DMEM supplemented with 10 mM pyruvate. To determine the basal and maximal respiration, carbonyl cyanide-4-(trifluoromethoxy)phenylhydrazone (FCCP, an inducer of maximal respiration; 0.25 μM) and antimycin A/rotenone (2 μM) were added to hepatocytes. The basal OCR was calculated by the OCR baseline levels before FCCP injection minus the average of three OCR levels after antimycin A/rotenone injection (non-mitochondrial respiration). Maximal OCR was produced by subtracting non-mitochondrial respiration from the OCR levels after FCCP injection^42^. The production and infusion of Ad-GFP and Ad-GFP-RORα1 were as previously described^15^.

### Real-time confocal microscopy

For the time course of experiments, hepatocytes were plated in DMEM with 10% FBS 4 h prior to refreshment of media or transduction. After 18 h, hepatocytes were cultured in Earle’s balanced salt solution with 5.5 mM glucose for 2 h. Next, cells were exposed to DMEM with 25 mM glucose and 0.3 mM palmitic acid conjugated with bovine serum albumin for the indicated time course. For real-time confocal microscopy, mitochondria were tagged by Ad-COX8a-GFP for visualization, as described previously^43^. Subsequently, live images were acquired at 37 °C using a confocal microscope with a Nikon Plan Apochromat 20×/0.75 objective (Nikon Eclipse Ti; Nikon, Japan). Ad-COX8a-GFP was kindly provided by Dr. Lee C-H (Harvard University, MA).

### Western blotting, ChIP, and quantitative real-time PCR (qRT–PCR)

Western blotting was performed as described previously using specific antibodies against α-SMA (ab7817, Abcam, 1:5000), TGFβ1 (sc-130348, Santa Cruz Biotechnology, 1:1500), Bnip3 (ab109362, Abcam, 1:7000), phospho-DRP1 (Ser616) (#4494, Cell Signaling Technology, 1:2000), Drp1 (#8570, Cell Signaling Technology, 1:2000), Fis1 (ab71498, Abcam, 1:2000), RORα (sc-6062, Santa Cruz Biotechnology, 1:2000), and actin (sc-1616, Santa Cruz Biotechnology, 1:2000). The ChIP assay was performed using anti-RORα (sc-6062, Santa Cruz Biotechnology), anti-p300 (sc-585, Santa Cruz Biotechnology), and anti-histone 3 (acetyl K27) (ab4729, Abcam) antibodies or a control IgG antibody (Santa Cruz Biotechnology), and specific primers (Supplementary Table)^19^. Relative mRNA expression was determined by qRT–PCR using the ABI StepOnePlus^TM^ Real-Time PCR system (Applied Biosystems, Foster City, CA) using specific primers (Supplementary Table). The mRNA expression of genes was calculated relative to controls using the 2^−ΔΔCT^ method^19^.

### Reporter gene analysis

The RORE/Bnip3-Luc and RORE(I)/PGC-1α-Luc were constructed by inserting three copies of putative ROREs into a pGL2-promoter vector (Promega) using specific oligomers (Supplementary Table). An eukaryotic expression vector encoding RORα1 ΔDBD was constructed by a PCR-mediated deletion method. Chang liver cells (CCL-13TM, ATCC, Rockville, MD) were transfected with a plasmid mixture containing reporter plasmid, eukaryotic expression vector, and pCMV-β-galactosidase using the Polyfect (Qiagen) according to the manufacturer’s protocol. Cells were lysed using luciferase lysis buffer (Promega, Madison, WI) and luciferase activity was measured using LB9508 luminometer (Berthold, Bad Wildbad, Germany) and normalized to β-galactosidase activity.

### Database-based gene expression analysis

Database-based gene expression analysis was conducted using public datasets obtained from GEO site at the NCBI gene expression (http://www.ncbi.nlm.nih.gov/geo/). The data from GSE33814 and GSE48452 processed as median-normalized signal intensity value. These public datasets were obtained from liver tissues of human patients. Normal (n = 13) and steatohepatitis (n = 12) for GSE33814^28^; healthy obese (n = 27) and NASH (n = 18) for GSE48452^27^.

### Statistical analysis

All analyses were performed using the GraphPad Prism software. Statistical analyses between two groups were conducted using the nonparametric Mann–Whitney *U* test (two-tailed, Figs 1, 2a, 3a right, 3b, 4a right, 4c, 5a) or unpaired Student’s t-test (two-tailed, Fig. 4f). Two-way ANOVA followed by the Bonferroni posttest was used to analyze the statistical significance of complex I activity (Fig. 2b), OCRs (Fig. 3a left, 4a left), and of *ex vivo* studies of nutrient switch (Figs 3c and 4b). Data are presented as the mean ± SEM. Statistical significance was set at *P* < 0.05.

## Electronic supplementary material

Supplementary Information
