# Supplementary material for: Liver-specific deletion of RORα aggravates diet-induced nonalcoholic steatohepatitis by inducing mitochondrial dysfunction
Source: Sci Rep. 2017 Nov 22;7:16041. doi: 10.1038/s41598-017-16077-y (PMC5700103; doi:10.1038/s41598-017-16077-y)
Supplement: Supplementary file 1 — Supplementary Information [file 41598_2017_16077_MOESM1_ESM.pdf]

Supplementary Information

Liver-specific deletion of RORα aggravates diet-induced nonalcoholic steatohepatitis by inducing mitochondrial dysfunction

Hyeon-Ji Kim[1], Yong-Hyun Han[1], Hyelin Na[1], Ju-Yeon Kim[1], Taewook Kim[2], Hye-Jin Kim[1], Chanseok Shin[2], Jung Weon Lee[1,4], and Mi-Ock Lee[1,3,4]

[1]College of Pharmacy, Seoul National University, Seoul, Korea; [2]Department of Agricultural Biotechnology, Seoul National University, Seoul, Republic of Korea; [3]Bio-MAX institute, Seoul National University, Seoul, Korea; [4]Research Institute of Pharmaceutical Sciences, Seoul, Republic of Korea

Supplementary Methods

RNA-seq and ChIP-seq analyses

The RNA used in sequencing analysis was obtained from the liver tissues of HFD-fed RORα-LKO and RORα$^{f/f}$ mice. For control and test RNAs, the construction of a library was performed using a SENSE mRNA-Seq Library Prep Kit (Lexogen, Inc., Austria), according to the manufacturer’s instructions. High-throughput sequencing was performed as paired-end 100 sequencing using HiSeq 2500 (Illumina, Inc., USA). RNA-Seq reads were mapped using the TopHat software tool, to obtain the alignment file. The alignment file was used for assembling transcripts, estimating their abundance, and detecting the differential expression

of genes or isoforms using cufflinks. Gene classification was based on searches of the BioCarta (http://www.biocarta.com/), GenMAPP (http://www.genmapp.org/), DAVID (http://david.abcc.ncifcrf.gov/), and Medline databases (http://www.ncbi.nlm.nih.gov/). The GO analysis was performed in DAVID Bioinformatics Resources 6.7. RORα ChIP-seq (GSE59486) and liver input control (GSE26345) were downloaded from gene expression omnibus (GEO) (http://www.ncbi.nlm.nih.gov/geo) [1, 2]. A public ChIP-seq is conducted in C57BL/6 mouse liver. Low-quality small RNA reads and adapter sequences were removed using FASTX-Toolkit (http://hannonlab.cshl.edu/fastx_toolkit/). Analysis was performed as previously described [2]. High-quality reads were aligned with the mouse reference genome (mm9) using Bowtie v1.1.2 with options '-n 2 -l 34 --best --strata -m 1' [3]. Only uniquely aligned reads to the genome were chosen for further analysis. Peak calling was performed using HOMER and a published liver input control [4]. Peaks were filtered according to two criteria: (1) score ≥ 10 and (2) peaks between –100 kb from transcription start site (TSS) and TSE. ChIP-seq peaks were visualized in IGV [5].

Analysis of mitochondrial mass using flow cytometry

Primary hepatocytes were isolated and incubated with low glucose DMEM media containing 100 nM MitoTracker green FM (Invitrogen) for 30 min. Green fluorescence was detected by flow cytometry.

Accession number

Raw data for HFD-fed control and RORα-LKO mouse RNA-seq are available in GSE90844.

Supplementary Table.

Gene	Accession number		Nucleotide sequence	Species	Purpose
RORα	NM_013646.2<br>NM_001289916.1	Sense<br>Antisense	5'-TTTCAGGAGAAGTCAGCAGAG-3'<br>5'-TCTGCTGGTCCGATCAATCAA-3'	Mouse	qRT-PCR primer
RORβ	NM_001043354.2<br>NM_146095.4<br>NM_001289921.1	Sense<br>Antisense	5'-ATGGCAGACCCACACCTACG-3'<br>5'-TATCCGCTTGGCGAACTCC-3'	Mouse	
RORγ	NM_011281.3<br>NM_001293734.1	Sense<br>Antisense	5'-CGAGATGCTGTCAAGTTTGGC-3'<br>5'-TGTAAGTGTGTCTGCTCCGCG-3'	Mouse	
LXRα	NM_001177730.1<br>NM_013839.4	Sense<br>Antisense	5'-AGGAGTGTCGACTTCGCAAA-3'<br>5'-CTCTTCTTGCCGCTTCAGTTT-3'	Mouse	
Fasn	NM_007988.3	Sense<br>Antisense	5'-CATGACCTCGTGATGAACGTGT-3'<br>5'-CGGGTGAGGACGTTTACAAAG-3'	Mouse	
SCD1	NM_009127.4	Sense<br>Antisense	5'-AGATCTCCAGTTCTTACACGACCAC-3'<br>5'-GACGGATGTCTTCTTCCAGGTG-3'	Mouse	
Acly	NM_001199296.1<br>NM_134037.3	Sense<br>Antisense	5'-GATGAAGTGGCACCTGCAAAG-3'<br>5'-GGTATGTCGGCTGAAGAGGGT-3'	Mouse	
Acaca	NM_133360.2	Sense<br>Antisense	5'-GCGGGAGGAGTTCCTAATTC-3'<br>5'-GGTTGGCATTGTGGATTTTC-3'	Mouse	
CD36	NM_001159558.1<br>NM_001159557.1<br>NM_001159556.1<br>NM_001159555.1<br>NM_007643.4	Sense<br>Antisense	5'-TCCTCTGACATTTGCAGGTCTATC-3'<br>5'-AAAGGCATTGGCTGGAAGAA-3'	Mouse	
TNFα	NM_013693.3	Sense<br>Antisense	5'-AATGGCCTCCCTCTCATCAGTT-3'<br>5'-CCACTTGGTGGTTTGCTACGA-3'	Mouse	
NLRP3	NM_145827.3	Sense<br>Antisense	5'-AGCCTTCCAGGATCCTCTTC-3'<br>5'-CTTGGGCAGCAGTTTCTTTC-3'	Mouse	
IL-1β	NM_008361.4	Sense<br>Antisense	5'-AGAGCCCATCCTCTGTGACTCA-3'<br>5'-TGCTTGGGATCCACACTCTCCA-3'	Mouse	
Col1a1	NM_007742.4	Sense<br>Antisense	5'-GACATCCCTGAAGTCAGCTGC-3'<br>5'-TCCCTTGGGTCCCTCGAC-3'	Mouse	
α-SMA	NM_007392.3	Sense<br>Antisense	5'-GGCACCACTGAACCCTAAGG-3'<br>5'-TCTCCAGAGTCCAGCACAAT-3'	Mouse	
TGFβ	NM_011577.2	Sense<br>Antisense	5'-CGACATGGAGCTGGTGA-3'<br>5'-TCCCGAATGTCTGACGTA-3'	Mouse	
MMP-2	NM_008610.3	Sense<br>Antisense	5'-TTTGCTCGGGCCTTAAAAGTAT-3'<br>5'-CCATCAAATGGGTATCCATCTC-3'	Mouse	
TIMP1	NM_001294280.2<br>NM_011593.2<br>NM_001044384.1	Sense<br>Antisense	5'-CTTGGTTCCCTGGCGTACTC-3'<br>5'-ACCTGATCCGTCCACAAACAG-3'	Mouse	
Bnip3	NM_009760.4	Sense<br>Antisense	5'-CTCCCAGACACCACAAGATAC-3'<br>5'-CTTCCTCAGACAGAGTGCTG-3'	Mouse	
Drp1	NM_152816.3<br>NM_001025947.2<br>NM_001276340.1<br>NM_001276341.1	Sense<br>Antisense	5'-CGTGACAAATGAAATGGTGC-3'<br>5'-CATTAGCCCACAGGCATCAG-3'	Mouse	
Fis1	NM_025562.3<br>NM_001163243.1	Sense<br>Antisense	5'-AGGCTCTAAAGTATGTGCGAGG-3'<br>5'-GGCCTTATCAATCAGGCGTTC-3'	Mouse	
Pink1	NM_026880.2	Sense<br>Antisense	5'-GCTTGCCAATCCCTTCTATG-3'<br>5'-CTCTCGCTGGAGCAGTGAC-3'	Mouse	
Mfn1	NM_024200.4	Sense<br>Antisense	5'-CCTACTGCTCCTTCTAACCCA-3'<br>5'-AGGGACGCCAATCCTGTGA-3'	Mouse	
Mfn2	NM_001285920.1<br>NM_133201.3<br>NM_001285921.1<br>NM_001285922.1<br>NM_001285923.1	Sense<br>Antisense	5'-AGAACTGGACCCGGTTACCA-3'<br>5'-CACTTCGCTGATACCCCTGA-3'	Mouse	

OPA1	NM_001199177.1	Sense	5'-CTGAGGCCCTTCTCTTGTTAGG-3'	Mouse	
		Antisense	5'-CTGACACCTTCCTGTAATGCTTG-3'		
PGC-1α	NM_008904.2	Sense	5'-CCCTGCCATTGTTAAGACC-3'	Mouse	
		Antisense	5'-TGCTGCTGTTCCTGTTTTC-3'		
TFAM	NM_009360.4	Sense	5'-GGAATGTGGAGCGTGCTAAAA-3'	Mouse	
		Antisense	5'-ACAAGACTGATAGACGAGGGG-3'		
Idha	NM_029573.2	Sense	5'-CCTCCTGCTTAGTGCTGTGA-3'	Mouse	
		Antisense	5'-CGTTGCCTCCCAGATCTTT-3'		
Acadm	NM_007382.5	Sense	5'-GATCGCAATGGGTGCTTTTGATAGAA-3'	Mouse	
		Antisense	5'-AGCTGATTGGCAATGTCTCCAGCAAA-3'		
ND1	NC_005089.1<br>NC_006914.1	Sense	5'-ACGCAAAATCTTAGGGTACA-3'	Mouse	
		Antisense	5'-GAGTGATAGGGTAGGTGCAA-3'		
COX1	NC_005089.1<br>NC_006914.1	Sense	5'-ATTCGAGCAGAATTAGGTCA-3'	Mouse	
		Antisense	5'-CTCCGATTATTAGTGGGACA-3'		
Ndufv1	NM_025523.1	Sense	5'-TTCCTCTGGATTCACCCTCA-3'	Mouse	
		Antisense	5'-CATGAGGAGCGCGAGTATTT-3'		
Sdha	NM_023281.1	Sense	5'-GAAAGGCGGGCAGGCTCATC-3'	Mouse	
		Antisense	5'-CACCACGGCACTCCCCATTTT-3'		
Uqcr	NM_025650.2	Sense	5'-TGCCGAGGCCTCAGACACAG-3'	Mouse	
		Antisense	5'-TCCAAGGCATAAGAATAAGGTTT-3'		
Cox5a	NM_007747.2	Sense	5'-TTGATGCCTGGGAATTGCGTAAAG-3'	Mouse	
		Antisense	5'-AACAACCTCCAAGATGCGAACAG-3'		
Atp5g1	NM_007506.6<br>NM_001161419.1	Sense	5'-AGTTGGTGTGGCTGGATCA-3'	Mouse	
		Antisense	5'-GCTGCTTGAGAGATGGGTTC-3'		
CytC	NM_007808.4	Sense	5'-GGAGGCAAGCATAAGACTGG-3'	Mouse	
		Antisense	5'-TCCATCAGGGTATCCTCTCC-3'		
18s rRNA	NR_003278.3	Sense	5'-GTAACCCGTTGAACCCCATT-3'	Mouse	
		Antisense	5'-CCATCCAATCGGTAGTAGCG-3'		
Reads-enriched region in Bnip3		Sense	5'-GAAACTGGCGTGATGAAATCTT-3'	Mouse	ChIP primer
		Antisense	5'-GATCTCACTTTGCAGCCAAC-3'		
Reads-enriched region in PGC-1α I		Sense	5'-CGGAGCTGCTAACTAACAATGG-3'	Mouse	
		Antisense	5'-GATGTGTATCACTGCACCACAC-3'		
II		Sense	5'-GTGTGGTGAAGAGTGAGGAT-3'	Mouse	
		Antisense	5'-AGGATTGCTATGAGCCTCTG-3'		
III		Sense	5'-GGCCCTGCCTGATCTTTAG-3'	Mouse	
		Antisense	5'-GAGCACACAAACAAGAAGTTAGG-3'		
RORα$^{f/f}$ allele (P1 and P2)		Sense	5'-TTGTGTATACCACCACAAGTGCACC-3'	Mouse	Genotyping primer
		Antisense	5'-AGTACAGGACACTTCGGTGTCTACC-3'		
Alb^{Cre} allele (Cre)		Sense	5'-TACAGACTGTGAGCAGATGTTC-3'	Mouse	
		Antisense	5'-TTCTTGCGAACCTCATCACTCGTTG-3'		
RORE/Bnip3		Sense	5'-CCCAACTGGGACGCCCAACTGGGACGC CCAACTGGGACGCG-3'		Cloning
		Antisense	5'-CTAGCGCGTCCCAGTTGGGCGTCCCAG TTGG GCGTCCCAGTTGGGAGCT-3'		
RORE(I)/ PGC-1α		Sense	5'-CGGGAGGGTCAAGTGGGAGGGTCAAGT GGGAGGGTCAAGTG-3'		
		Antisense	5'-CTAGCACTTGACCCTCCCACTTGACCC TCCCACTTGACCCTCCCGAGCT-3'		

Supplementary Figures

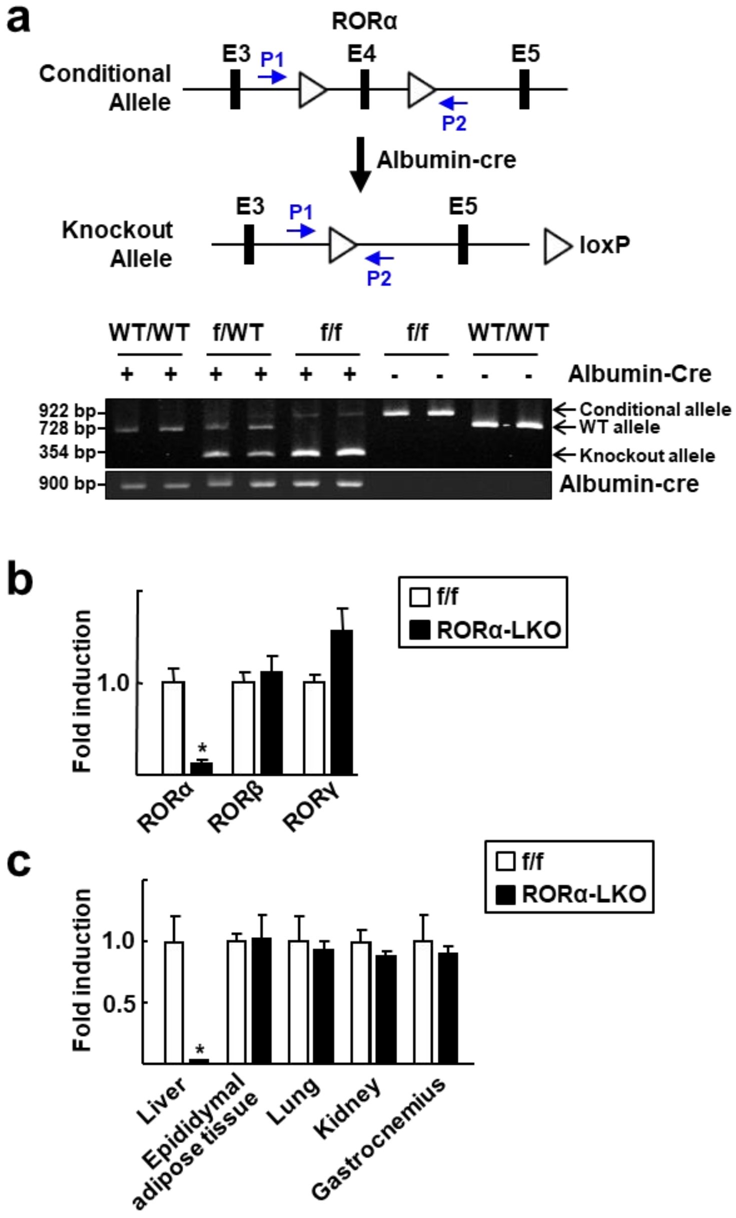

Figure S1. Generation and characterization of the liver-specific RORα knockout mice.
(a) Schematic representation of the conditional RORα floxed allele and the strategy of generation of the liver-specific RORα-deficient allele through Albumin-Cre recombination. E; Exon (upper). Genotyping analysis of the RORα$^{f/f}$ and Alb^{Cre}-RORα$^{f/f}$ (RORα-LKO) mice. The primer sequences (P1/P2 and Cre) used for the genotyping were shown in Table (lower).
(b) The mRNA expression levels of the RORα, RORβ, and RORγ in liver tissues were measured by qRT-PCR. Values represent mean ± SEM. $^{*}P < 0.05$ *vs* RORα$^{f/f}$ (n=4).
(c) The relative mRNA expression levels of RORα in various tissues. Values represent mean ± SEM. $^{*}P < 0.05$ *vs* RORα$^{f/f}$ (n=4).

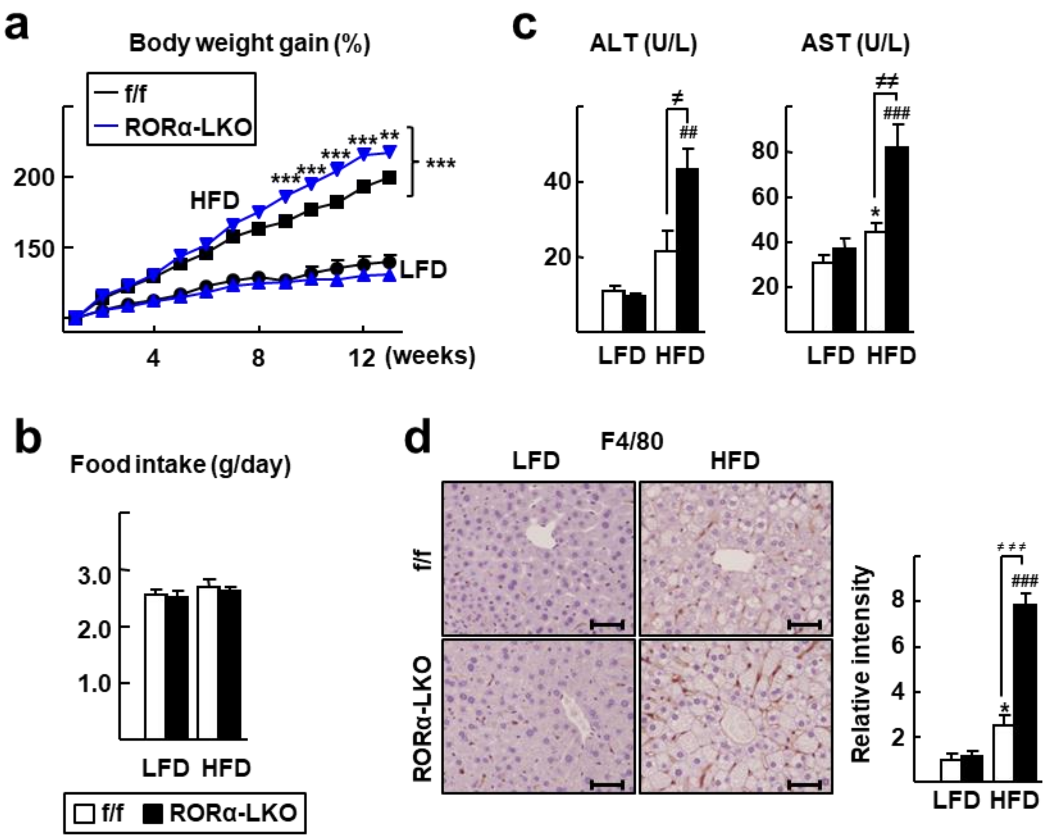

Figure S2. Liver-specific KO of RORα exacerbates the HFD-induced body weight gain and liver injury.

(a) Six week-old RORα-LKO and RORα$^{f/f}$ mice were fed with either LFD or HFD for 12 weeks. Body weight gain (%) represents the % increase compared to initial body weight (ranged from 17.0g to 23.6g). Data represent as mean ± SEM. $^{**}P < 0.01$, $^{***}P < 0.001$ *vs* HFD-fed RORα$^{f/f}$ (n=7-9).

(b) Food intake (g/day). Data represent as mean ± SEM.

(c) Serum ALT and AST activities were measured by standard clinical chemistry assays at the end of experiments. Data presented as mean ± SEM (n=7-9). ALT; $^{\#\#}P < 0.01$ *vs* LFD-fed RORα-LKO, $^{\neq}P < 0.05$ *vs* HFD-fed RORα$^{f/f}$, AST; $^{*}P < 0.05$ *vs* LFD-fed RORα$^{f/f}$, $^{\#\#\#}P < 0.001$ *vs* LFD-fed RORα-LKO, $^{\neq\neq}P < 0.01$ *vs* HFD-fed RORα$^{f/f}$.

(d) Histological staining of F4/80. Scale bar: 50 μm. Representative Images of liver sections from the RORα$^{f/f}$ and RORα-LKO mice were presented. Relative intensities were quantified using ImageJ. Values represent mean ± SEM (n=6-8). $^{*}P < 0.05$ *vs* LFD-fed RORα$^{f/f}$, $^{\#\#\#}P < 0.001$ *vs* LFD-fed RORα-LKO, $^{\neq\neq\neq}P < 0.001$ *vs* HFD-fed RORα$^{f/f}$.

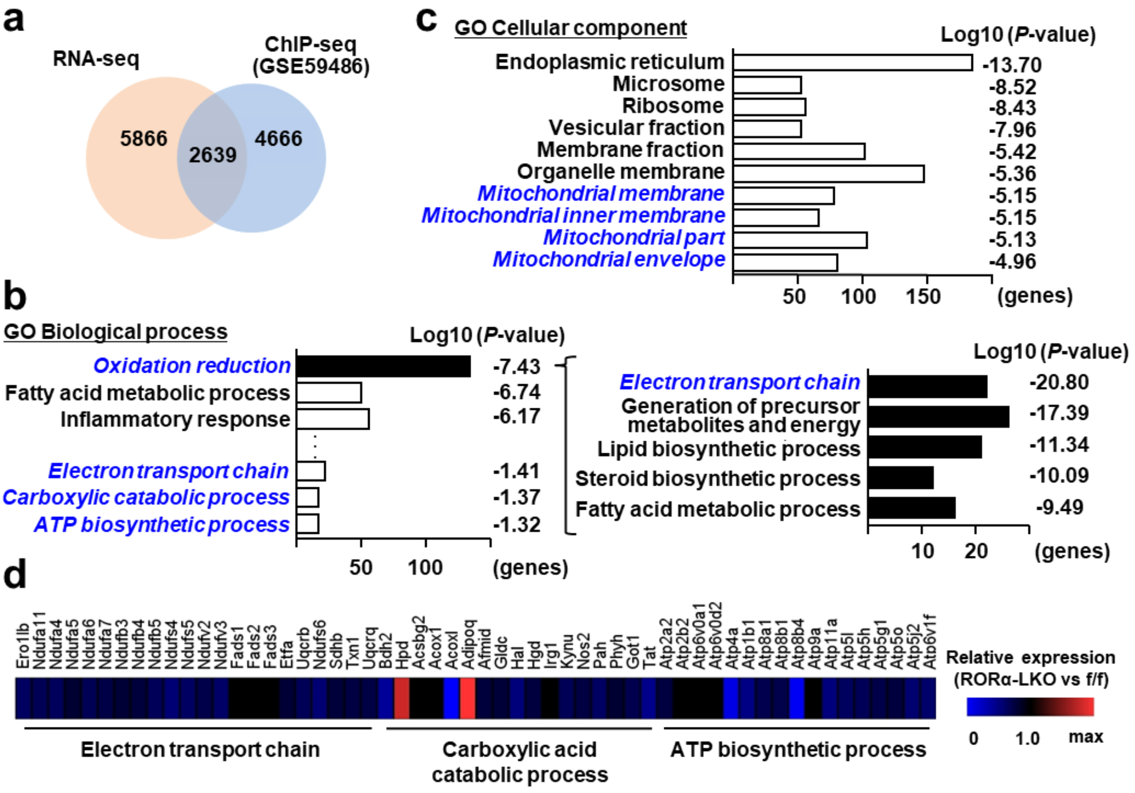

Figure S3. Gene expression profiling for liver tissues from the RORα-LKO mice.

(a) Venn diagrams showing the overlapping target genes those delineated by the RNA-seq analysis in this study and the publically available ChIP-seq dataset (Fang *et al*, 2014). RNA-seq data was obtained from liver tissues of RORα-LKO and RORα$^{f/f}$ mice and the fold change of each gene was calculated by dividing the normalized read count of HFD-fed RORα-LKO by that of RORα$^{f/f}$. (cut-off: 0.8-fold, 1.5-fold).

(b) GO biological process analysis for the overlapped genes (left). Genes clustered in the top ranked GO biological process term, oxidation reduction, were further analyzed for GO biological process analysis. Top 5 GO based on P-value are shown (right).

(c) GO cellular component analysis for the overlapped genes. Bar graph showing the top 10 enriched cellular component based on P-value. The number of genes in each ontology category is shown.

(d) Heat map representation of differentially regulated genes associated with electron transport, carboxylic acid catabolic process, and ATP biosynthetic process. Gene symbols are indicated.

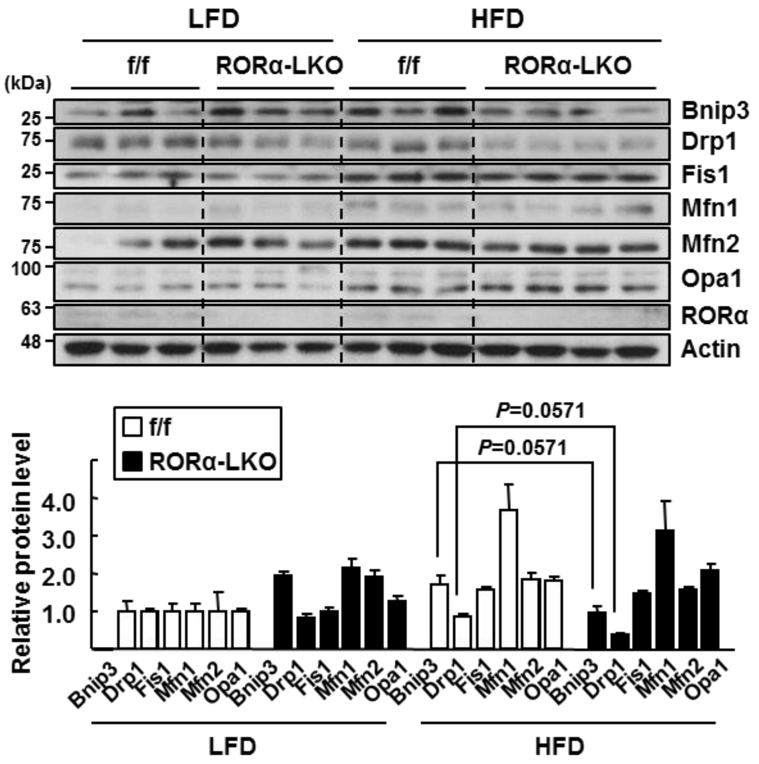

Figure S4. The expression levels of Bnip3 and Drp1 were decreased in the HFD-fed RORα-LKO with marginal significances.

The levels of indicated proteins were analyzed by western blotting (upper). Band intensities of each protein were quantified using ImageJ and normalized to that of actin (lower). Data presented as mean ± SEM (n=3-4).

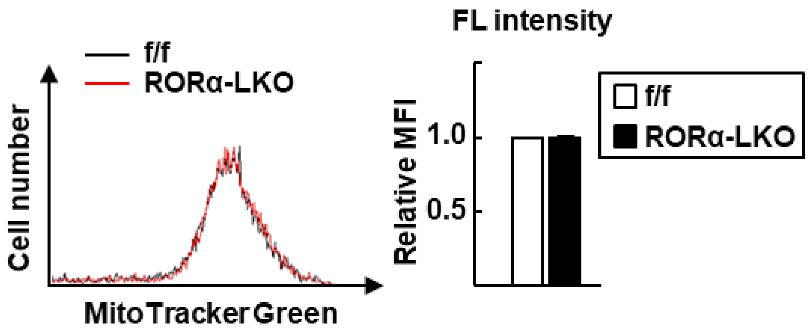

Figure S5. Analysis of mitochondrial mass using flow cytometry.

Primary hepatocytes were isolated from control and RORα-LKO mice and were incubated with 100nM MitoTracker Green FM (Invitrogen) for 30 min. Green fluorescence was detected by flow cytometry (left) and relative mean fluorescence intensity (MFI) of stained cells was presented. Data presented as mean ± SEM (n=3).

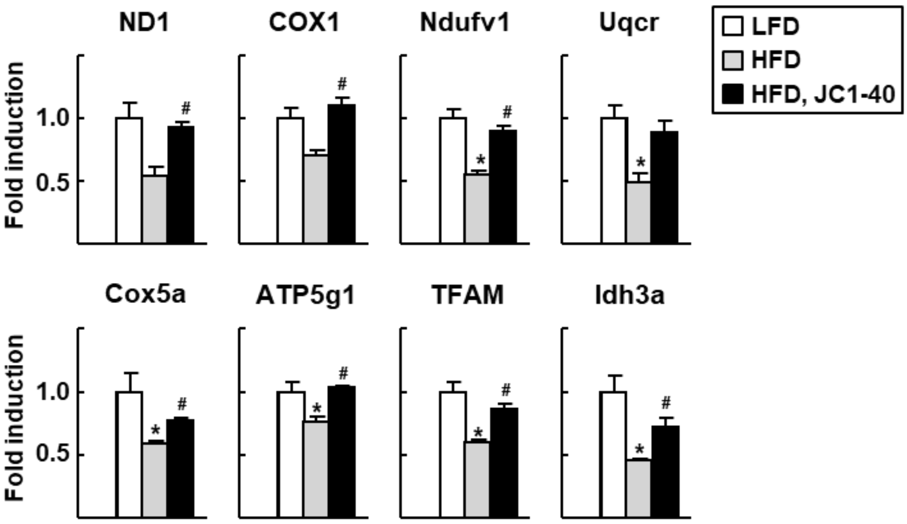

Figure S6. Administration of JC1-40 restores the expression level of mitochondrial OXPHOS genes in the HFD-fed mice.

Six weeks-old C57BL/6 mice were fed with either LFD or HFD for 12 weeks. After 7 weeks, JC1-40 was administered daily at doses of 5 mg/kg/day by oral gavage for 5 weeks. The expression levels of mitochondrial OXPHOS and genes related to mitochondrial biogenesis were analyzed by qRT-PCR. Data presented as mean ± SEM (n=4). $^{*}P < 0.05$ *vs* LFD-fed mice and $^{\#}P < 0.05$ *vs* HFD-fed mice. ND1, NADH-ubiquinone oxidoreductase chain 1; COX1, Cytochrome c oxidase 1; Ndufv1, NADH dehydrogenase [ubiquinone] flavoprotein 1, mitochondrial; Uqcr, ubiquinol-cytochrome c reductase, complex 3 subunit 11; Cox5a, Cytochrome c oxidase subunit 5a; ATP5g1, ATP synthase lipid-binding protein, mitochondrial; TFAM, Mitochondrial transcription factor A; Idh3a, Isocitrate dehydrogenase [NAD] subunit alpha, mitochondrial.

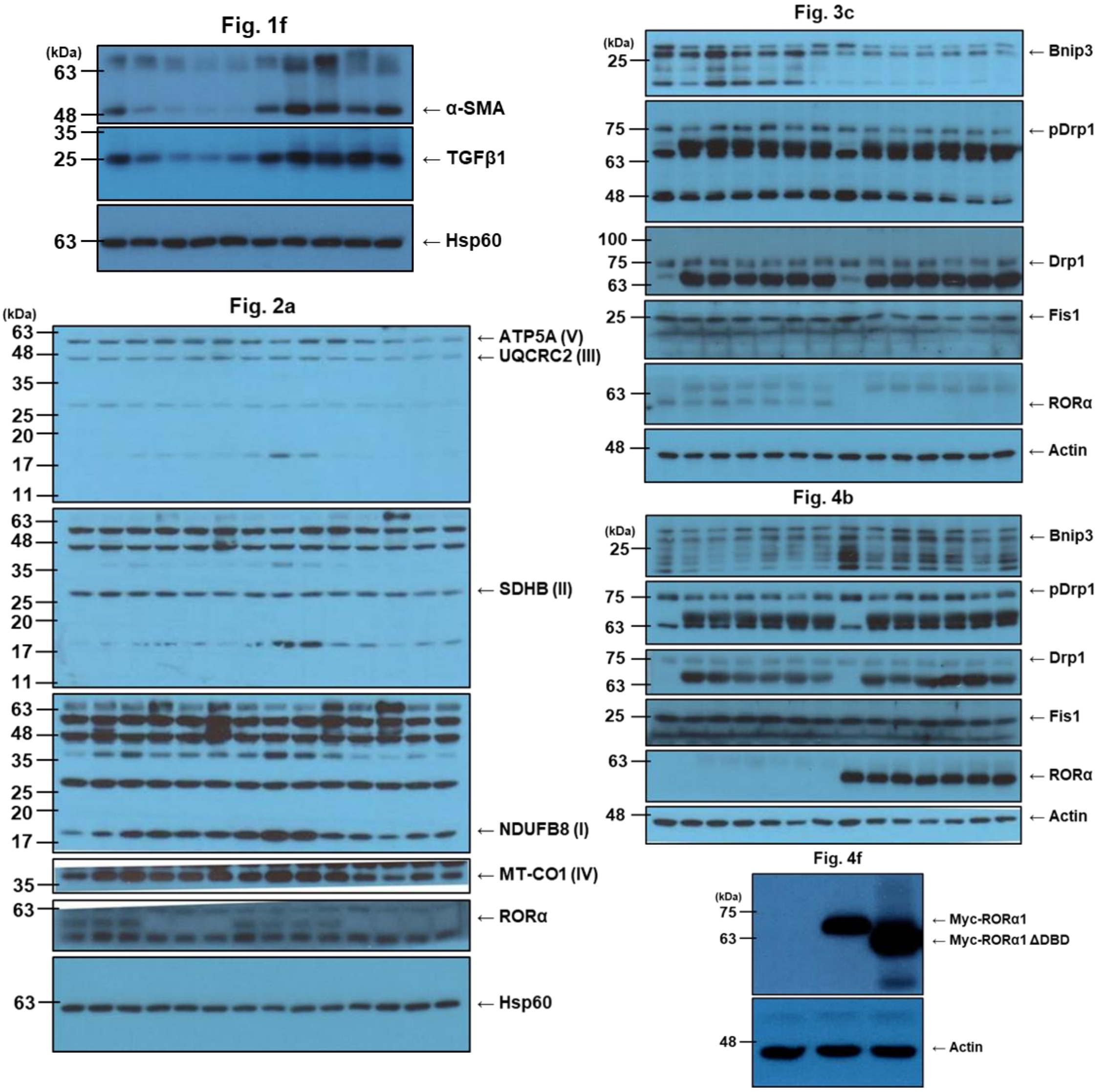

Figure S7. The original images of blots presented in the main figures.

The original images of the main figures are presented and the numbers in the left side of the blot indicate the molecular mass of each protein.

References

1. Fang, B. *et al*. Circadian enhancers coordinate multiple phases of rhythmic gene transcription in vivo. *Cell* **159,** 1140-1152 (2014).

2. Feng, D. *et al*. A circadian rhythm orchestrated by histone deacetylase 3 controls hepatic lipid

metabolism. *Science* **331,** 1315–1319 (2011).

3. Langmead, B., Trapnell, C., Pop, M. & Salzberg, S. L. Ultrafast and memory-efficient alignment of short DNA sequences to the human genome. *Genome Biol.* **10,** R25 (2009).

4. Heinz, S. *et al*. Simple combinations of lineage determining transcription factors prime cis-regulatory elements required for macrophage and B cell identities. *Mol. Cell* **38,** 576–589 (2010).

5. Robinson, J. T. *et al*. Integrative genomics viewer. *Nat. Biotechnol.* **29,** 24–26 (2011).
